# Integrative Postural Rehabilitation for Kyphotic Deformity in a Patient with Parkinson’s Disease: A Case Report and Literature Review

**DOI:** 10.3390/jcm14113705

**Published:** 2025-05-25

**Authors:** Ye-Rim Yun, Ji-Sung Yeom, Joon-Seok Lee, Doori Kim, Yoon Jae Lee, In-Hyuk Ha, Do-Young Kim

**Affiliations:** 1 Department of Acupuncture & Moxibustion, Jaseng Korean Medicine Hospital, Seoul 06110, Republic of Korea; yyl0531@jaseng.co.kr (Y.-R.Y.); wltjd153@jaseng.co.kr (J.-S.Y.); paraojs@jaseng.co.kr (J.-S.L.); 2 Jaseng Spine and Joint Research Institute, Jaseng Medical Foundation, Seoul 06110, Republic of Korea; doori.k07@gmail.com (D.K.); goodsmile8119@gmail.com (Y.J.L.); hanihata@gmail.com (I.-H.H.); 3 Department of Public Health Science, Seoul National University, Seoul 08826, Republic of Korea

**Keywords:** postural rehabilitation, integrative medicine, exercise, kyphotic deformity, Parkinson’s disease

## Abstract

Spinal deformities, particularly thoracolumbar kyphosis, affect approximately one-third of patients with Parkinson’s disease (PD) and significantly impair their quality of life and mobility. Conventional treatments, including levodopa and surgical interventions, have limited efficacy, necessitating alternative therapies. In this report, a 76-year-old woman with PD and severe thoracolumbar kyphosis (TK: 77.7°; sagittal vertical axis [SVA]: 95.55 mm) experienced postural instability and gait impairment. She underwent integrative postural rehabilitation (acupuncture, pharmacopuncture, Chuna spinal manual therapy, thermotherapy, and bodyweight exercises). A 4-week inpatient treatment improved spinal alignment (TK: 61.1°; SVA: 77.84 mm), gait, postural stability (MDS-UPDRS score improved by 3 points), and functional outcomes, with reductions in the Oswestry Disability Index (70 to 31) and pain severity (Numeric Rating Scale: 50 to 40). No adverse events were observed. Integrative postural rehabilitation can mitigate paraspinal muscle atrophy and fatty infiltration by promoting protein synthesis, neurotrophic factor expression, and proprioceptive neuromodulation. Our literature review suggests that proprioceptive stimulation and exercise enhances postural stability and gait, aligning with the outcomes of this case. This report suggests that integrative rehabilitation may improve kyphotic deformities and related motor dysfunctions in patients with PD. Further research is warranted to validate the treatment’s efficacy and long-term benefits.

## 1. Introduction

Patients with Parkinson’s disease (PD) experience spinal deformities, characterized by degenerative axial alignment abnormalities, in approximately one-third of cases [1,2]. These deformities significantly impair the quality of life (QoL) of patients with PD by adversely affecting their walking ability, postural stability, social functioning, and mood regulation [3,4]. Postural instability and gait disturbances, which are closely associated with these spinal changes, are the primary contributors to falls in patients with PD, occurring in 64% of patients [5]. This fall rate is double that of the general population aged 65 years and older, imposing a significant social burden on PD patients [6]. In addition, the increased severity of postural instability and motor dysfunction in patients with PD correlates with poor prognosis and comorbidities, such as cognitive impairment and depression [7].

The pathophysiology of spinal deformities in PD remains poorly understood, posing challenges for diagnostic and therapeutic strategies [8]. Current research suggests that these deformities result from dopamine-mediated dysfunction of the basal ganglia, which induces myopathy of the spinal support muscles [9]. However, the standard PD treatment with levodopa has shown limited efficacy in the management of spinal deformities [10]. Surgical interventions such as deep brain stimulation and spinal internal fixation have been debated for their effectiveness and safety, with an 86% reoperation rate reported [11,12].

Given the degenerative nature of PD, conservative treatments, including exercise, acupuncture, and spinal manipulation, are commonly used to manage spinal disabilities in clinical practice [13]. In Korean Medicine, combined therapy involving acupuncture and exercise has shown promise in improving pain and the range of motion (ROM) in degenerative axial conditions such as spinal stenosis and spondylosis [14]. However, rehabilitative approaches using Korean Medicine to treat spinal deformities and related symptoms in patients with PD remain underexplored.

This study presents a case of PD with spinal deformity in which a significant improvement was observed, particularly in the correction of thoracic hyperkyphosis. Integrative postural rehabilitation involving exercise and treatment based on Korean Medicine may offer a non-surgical and effective treatment alternative for improving thoracolumbar deformities and related symptoms in patients with PD.

## 2. Case Presentation

### 2.1. Patient Characteristics

A 76-year-old Asian woman diagnosed with PD in 2020 was admitted to the Jaseng Hospital of Korean Medicine on 19 August 2024, presenting with a forward bending posture accompanied by thoracic and lumbar spinal pain. The patient had a spouse and two adult children, all of whom were actively involved in her care and provided continuous support throughout her treatment. Comorbidities included hypothyroidism (diagnosed in 1980), diabetes and hyperlipidemia (2021), fatty liver (2022), atrophic gastritis (2022), and osteoporosis (2023). Details of the medication content are provided in Table A1. She had no history of spinal or joint surgery and maintained non-smoking and non-drinking habits. Routine blood and urine tests revealed no significant abnormalities, except for mildly elevated fasting blood sugar levels, which were attributed to diabetes.

**Figure 1:**
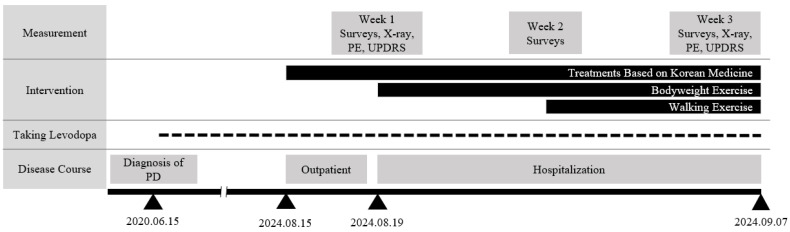
Timeline from PD onset to discharge after treatment. The duration of the treatment interventions is represented by the length of the horizontal bar. Radiographs were obtained at 1 and 3 weeks. The survey included NRS, ODI, and EQ-5D scores. The NRS score for spinal pain, ODI score for lower back pain, and EQ-5D score for quality of life were measured weekly. UPDRS for posture and gait and ROM for thoracolumbar movement by physical examination were measured at 1 and 3 weeks. EQ-5D, EuroQol-5 Dimension; NRS, Numeric Rating Scale; ODI, Oswestry Disability Index; PD, Parkinson’s disease; PE: Physical examination; ROM, range of motion; UPDRS, Unified Parkinson’s Disease Rating Scale.

The patient experienced thoracolumbar pain since the forward bending deformity occurred, which worsened and was accompanied by radicular leg pain in early 2024 after lifting a heavy object, prompting hospitalization. She could walk independently but required a cane or walker for support (Figure 1).

### 2.2. Diagnosis & Outcome Measurement

**Figure A1:**
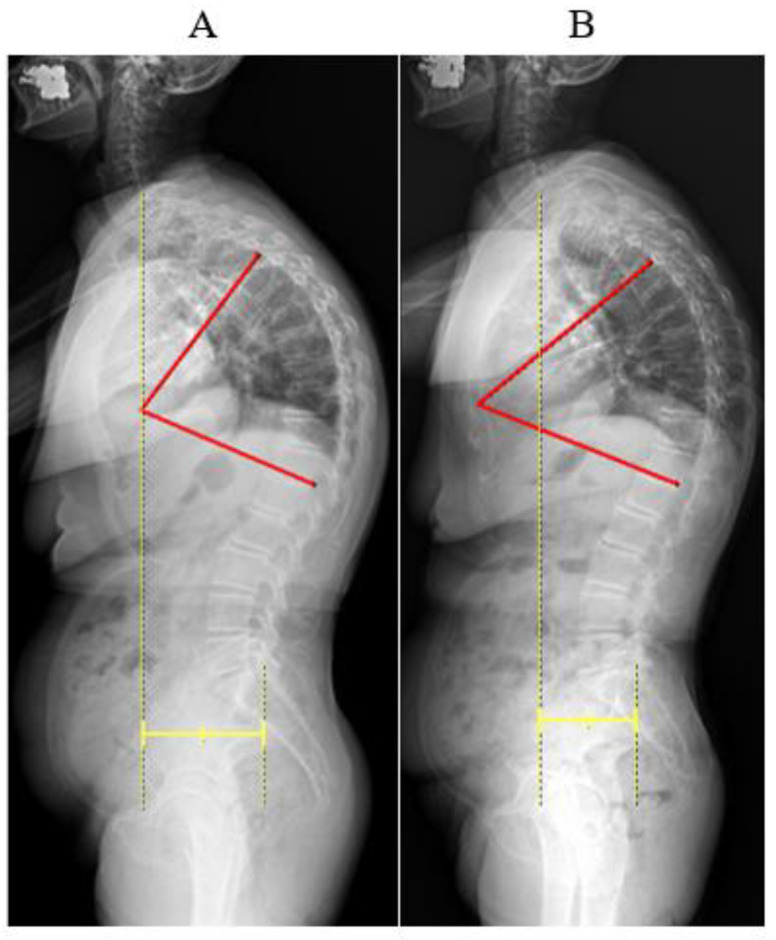
Metrics for measuring radiologic indicators in spinal alignment (**A**) before and (**B**) after treatment. The red line shows how the thoracic kyphotic angle (TK) is measured, and the yellow line shows how the distance between the sagittal vertical axis (SVA) is measured.

Along with the diagnosis of PD in 2020, thoracolumbar deformity was assessed using sagittal radiography at admission and discharge. Prior to radiography, the patient was instructed to standardize their posture by standing on an identical platform, maintaining a forward gaze, and holding their handles at the same height. The radiographic indicators measured included thoracic kyphosis (TK), defined as the angle between the upper endplate of T5 and the lower endplate of T12, and the sagittal vertical axis (SVA), defined as the distance between the vertical line of C7 and the superior posterior corner of S1 [15]. In the general population of women in their 70s, the average TK angle is 30° and the SVA distance is 30 mm [16], whereas the patient had values of 77.7° and 95.55 mm, respectively. (Figure A1).

In addition to radiological measurements, physical examinations and patient-reported outcome surveys were performed to evaluate physical function and pain. Gait and postural abnormalities were assessed at admission and discharge using the Movement Disorder Society-Unified Parkinson’s Disease Rating Scale (MDS-UPDRS) subscales, with a total of five items with a score range of 0–20 (higher = worse), including walking and balance, gait, freezing of gait, postural stability, and posture [17]. ROM was assessed spontaneously. Weekly evaluations of spinal disorder-related disability, pain, and QoL were conducted using the Numeric Rating Scale (NRS), Oswestry Disability Index (ODI), and EuroQol-5 Dimension (EQ-5D) surveys, and the scores were converted to a 0–100 scale (higher = worse) [18,19].

### 2.3. Interventions

Routine conservative treatments based on Korean Medicine clinical practice guidelines were implemented in accordance with back pain [20] (Table 1). The interventions included acupuncture, pharmacopuncture, manual spinal therapy, cupping, and thermal therapy. Treatments based on Korean Medicine were practiced once daily in outpatient clinics and twice daily during the hospitalization period. PD symptom management with levodopa was continued.

Acupuncture treatment was performed using 0.30 × 60 mm needles (30 gauge, DongBang Co., Seoul, Korea) on specific acupoints and muscles in addition to the guidelines [20]. GV16, GV20, GB20, and GB34 were targeted to activate dopaminergic neuromodulation within the brain [21,22]. Additional acupuncture and pharmacopuncture targeted the Ashi points in the thoracic and lumbar spine for tension and pain relief. Needling with electrical stimulation (20 Hz, 15 min) was applied to stimulate paraspinal muscles, addressing trunk bending in PD [11,23]. Chuna spinal manual therapy (15 min daily) was adapted to realign the spine and to promote passive joint movements [24].

Bodyweight exercises consisted of core muscle and thoracic mobility exercises. The core exercises focused on contracting and relaxing the abdominal muscles, deep erector spinae, and gluteus maximus, whereas the thoracic mobility exercises engaged the external oblique and serratus anterior. The exercises were performed daily for 20 min, progressing gradually in intensity (Table 2 and Table A2). In addition, walking exercises in the hospital corridor began after the back pain improved, with the patient wearing a pelvic girdle for gluteus maximus support. The walking distance and speed gradually increased.

### 2.4. Course of Symptoms and Outcomes

**Figure 2:**
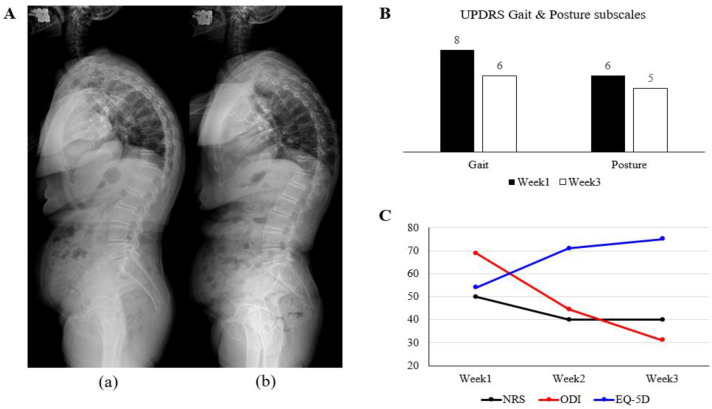
Progression of symptoms. (**A**) Whole-spine radiographic images before and after treatment. (**B**) Pre- and post-treatment questionnaires with subscales extracted from the UPDRS for gait and posture. (**C**) Numeric rating scale (NRS) for thoracolumbar pain, Oswestry Disability Index (ODI) for lumbar spine function, and EuroQol-5Dimension (EQ-5D) as a health-related quality of life instrument at weeks 1, 2, and 3 of the treatment periods.

Radiographic indicators demonstrated significant improvement in spinal alignment, with TK decreasing from 77.7° to 61.1° and SVA reducing from 95.55 to 77.84 mm at discharge (Figure 2A, Table 3). The gait and posture scores improved by 2 and 1 point(s), respectively (Figure 2B). Initially, the patient required assistive devices to walk and exhibited severe trunk flexion. Upon discharge, the patient walked independently with an improved posture. Back pain decreased (NRS: 50 to 40) with thoracolumbar ROM improvement in extension and lateral bending, and functional outcomes significantly improved (ODI: 70 to 31; EQ-5D: 54 to 75) (Figure 2C, Table 3). In particular, notable progress was observed in walking posture, walking distance, and personal hygiene activities, such as washing and dressing. No adverse effects were reported during hospitalization.

### 2.5. Review of Literature

A literature survey was conducted using a single database (PubMed) to identify clinical trials using rehabilitation interventions to treat postural instability in patients with PD and spinal deformities up to December 2024. The inclusion criteria were as follows: (1) studies including patients with PD and spinal deformity problems; (2) studies that had a randomized controlled trial (RCT) design and employed non-surgical rehabilitation programs; and (3) studies assessing changes in postural stability using relevant measurement tools. The main search terms used included “Parkinson’s disease,” “deformity,” “posture,” and “rehabilitation,” and references were reviewed for titles, abstracts, and full texts.

Of the 109 articles initially identified, three RCTs were ultimately included. The characteristics of these articles are summarized in Table 4. All the RCTs showed significant improvement in postural stability after a postural rehabilitation program that particularly stimulated proprioception [25,26,27]. However, the improvement in gait-related motor function was not statistically significant.

## 3. Discussion

Forward bending deformity of the spine in patients with PD, also known as camptocormia, is diagnosed when anterior flexion of the thoracolumbar spine exceeds 45° in the standing position, showing an increased TK angle and SVA in radiology [28]. The etiology of spinal deformities in PD remains unclear, but it has been suggested that proprioceptive control dysfunction resulting from basal ganglia degeneration leads to myopathy in the axial muscles [11,29].

Pathological changes, including atrophy and fatty degeneration of the paraspinal muscles, such as the multifidus, contribute to anterior spinal curvature [30]. Notably, it has been shown that patients with less muscle degeneration and a larger spinal multifidus cross-sectional area have a positive prognosis [11]. Moreover, weakness of the abdominals and gluteus muscles with tensors in thoracolumbar erectors follows the deformity, making it aggravated and, conversely, causes spinal deformation [31]. Current treatments include spinal surgery with fixation, whereas medication, deep brain stimulation, or exercise for proprioceptive restoration have shown limited effectiveness in treating deformities [10,29].

In this case, we prospectively employed treatments based on Korean Medicine for tissue release, neural stimulation, and restoration of muscle atrophy using minimally invasive interventions, anticipating a synergistic effect with exercise therapies. In terms of muscle tissue release, acupuncture and pharmacopuncture were applied to trigger tender points and reduce muscle tension, along with analgesic effects [32]. As shown in Figure 2C, physical function measured using ODI, such as activities involving walking or lifting stuff, improved and was accompanied by a reduction in soft tissue tension [33]. Although the pain severity was alleviated, it was sustained at an NRS score of 4. This is thought to be due to potential disorders that cause pain, such as degenerative changes in the lumbar spine and narrowed intervertebral disc space on radiography (Figure 2A).

Additionally, GV16, GV20, and GB20 were applied for acupuncture to stimulate the cortical areas responsible for gait and postural balance [34]. Stimulation of GB34, known to enhance neural responses in the substantia nigra by facilitating dopamine reabsorption, has been shown to improve motor function in patients with PD by activating the motor cortex via the basal ganglia circuit [35,36]. As a result, the TK angle was reduced, as shown in Figure 2A, and the patient was able to correct her posture to a normal posture and walk independently without a walker or support, showing improvement in the scores for posture and gait, as shown in Figure 2B.

Deep paraspinal muscles, which are key stabilizers involved in torso rotation and tilt, play a crucial role in degenerative spinal deformities, particularly in myopathies such as atrophy and increased fatty infiltration [37]. Acupuncture, with or without electronic stimulation, is thought to prevent muscle atrophy and fatty infiltration by promoting protein synthesis, fiber regeneration, and neurotrophic factor expression [23,38]. Additionally, acupuncture may inhibit α-synuclein, a key factor in PD pathogenesis, thereby preventing mitochondrial dysfunction in muscle tissues and regulating acetylcholine’s release at neuromuscular junctions [39,40]. Through musculoskeletal function improvement, the ODI and EQ-5D scores indicated enhanced personal hygiene (e.g., washing and dressing) and gait, requiring complex axial coordination (Figure 2C).

Chuna manual spine therapy and heat therapy were administered (Table 1). Spinal manual therapy directly influences muscle tone and thoracic kyphosis angle, promoting passive spinal movement and restoring ROM (Table 3) [41,42]. Complementarily, thermotherapy enhances peripheral circulation, potentially improving blood supply and immune cell activation [43]. Collectively, these therapies may synergistically facilitate paraspinal muscle function by relieving tension, promoting passive movement, and nourishing soft tissues [44].

RCTs were reviewed to further assess the efficacy of postural rehabilitation in patients with PD and spinal deformities (Table 4). All three RCTs employing proprioceptive sensory stimulation reported significant improvements in kyphotic curvature [25,26,27]. However, only one RCT that combined exercise with sensory stimulation demonstrated improvements in balance and gait [25]. The two other RCTs that used proprioceptive stimulation alone did not show significant gait improvement [26,27]. These findings suggest that combining sensory stimulation with exercise may enhance neuromuscular coordination, thereby improving posture, balance, and gait [45]. Acupuncture and electronic stimulation enhance proprioception by stimulating muscle spindles and sensory receptors, while Chuna therapy and thermal stimulation support peripheral circulation and tissue recovery [46,47]. In this context, together with exercises, these treatments based on Koran Medicine may synergistically restore both gait and address kyphotic deformity. Although further evidence considering symptom severity, demographic features, and standardized intervention modalities is needed, our study demonstrated treatment duration and outcomes comparable to those of the interventions reviewed in the literature. This approach may offer potential advantages over conventional therapies in terms of time efficiency, cost-effectiveness, and therapeutic benefit (Table 4).

This case demonstrates that integrative postural rehabilitation improves axial muscle weakness, alleviates thoracolumbar kyphosis, and stabilizes posture and gait. However, this study had some limitations. First, the lack of post-discharge follow-up data limits conclusions regarding treatment durability. Second, as the patient continued PD medication, its potential influence on the outcomes could not be excluded. Third, no objective imaging, such as magnetic resonance imaging, was available to confirm soft tissue changes, including fatty infiltration or muscle atrophy. Despite these limitations, this is the first reported case of rapid improvement in thoracolumbar kyphosis using exercise combined with treatment based on Korean Medicine. While the effects of individual treatments such as acupuncture, spinal manipulation, and exercise on postural rehabilitation have been previously reported, this study emphasizes the potential synergistic effects of their combination and proposes a novel therapeutic approach for neuro-degenerative disorders such as PD. Further well-designed clinical trials focusing on proprioceptive function outcomes as the primary endpoint are required to examine the proprioceptive impact of integrative postural rehabilitation for broader clinical applications.

## Figures and Tables

**Table 1:** Treatments based on Korean Medicine.

Intervention	Details
Acupuncture	GV16, GV20, GB20, GB34 Ashi points in back pain Trigger points of erector spinae (thoracic and lumbar region), sacrotuberous ligament, gluteus maximus, gluteus medius, gluteus minimus, iliopsoas muscle, biceps femoris, gastrocnemius, quadratus lumborum and sternocleidomastoid
Pharmacopuncture	Shinbaro2 ^A^ intramuscular injections
Electroacupuncture	Both sides of GB20 Paraspinal muscles
Chuna spinal manual therapy	Joint mobilization therapy Joint distraction therapy Fascia (soft tissue) therapy Joint manipulation therapy Craniosacral therapy
Other treatment	Moxibustion, cupping, infrared therapy, hot pack therapy

^A^ https://www.tandfonline.com/doi/full/10.2147/JPR.S413512 (accessed on 22 May 2025).

**Table 2:** Bodyweight Exercise Program.

Contents		Details	Intensity	Time
Core muscle exercises	Abdominal breathing	While keeping the knees bent, breathe in deeply, slowly expanding the abdomen without using the chest or neck muscles. Then, exhale slowly, pulling the abdomen in as you fully release the breath.	Without causing pain for 5–10 s	20 min
	Deep erector spinae strengthening	While maintaining abdominal breathing in a prone position, move with the feeling of lifting the hips and deep lower back towards the head as you exhale.		
	Gluteus maximus strengthening	While lying in a prone position, bend one knee and, using only the glute muscles, lift your leg slightly as you exhale. Hold this position for 5 s.		
Thoracic mobility exercises	External oblique abdominal muscle strengthening	Sit with feet flat on the ground and pelvis in a neutral position. Then, clasp hands behind the head and rotate only the upper body left and right.	Increase intensity by count up to 30	20 min
	Serratus anterior strengthening	Sit with feet flat on the ground and pelvis in a neutral position. Then, extend arms straight in front of the chest, clasp hands together, and rotate only the upper body left and right.		

**Table 3:** Angle Changes in Radiographic and Functional Assessments.

	Radiographic Index		Range of Thoracolumbar motion (degree)			
	TK (°)	SVA (mm)	Flexion	Extension	Lat. Bending (Rt./Lt.)	Rotation (Rt./Lt.)
Week 1	77.7	95.55	60	10	10/10	45/45
Week 3	61.1	77.84	60	20	15/15	45/45

SVA, Sagittal vertical axis (the distance between the C7 plumb line and the S1 superior posterior corner); TK, thoracic kyphosis (T5-12).

**Table 4:** Characteristics of the studies in the literature review.

Target Deformity [reference]	N. of Participants (Female, Mean Age [years])	Intervention (Control)	Intervention Category (period)	Measurement	Result
Thoracic bending [25]	20 (9, 69.1)	(1) GPR (2) GPR + KT (no treatment)	Exercise, sensory stimulation (4 weeks) Bracing (4 weeks)	(1) Angle of anterior and lateral bending of the trunk (2) BBS, TUG	(1) Angle of anterior bending: *p* < 0.01, angle of lateral bending: NS (2) BBS, TUG: *p* < 0.01
Thoracic and lumbar bending [26]	20 (8, 72.5)	Su-Per (physical therapy)	Sensory stimulation (4 weeks)	(1) Kyphosis and lordosis angles (2) Steps cadence, stride length, normalized velocity	(1) Kyphosis angle: *p* < 0.009, Lordosis angle: NS (2) Gait measurement: NS
Thoracic bending [27]	24 (9, 67.5)	Equistasi^®^ (usual care)	Sensory stimulation (8 weeks)	(1) Postural analysis during the gait cycle (2) UPDRS (gait scale)	(1) Trunk forward Bending: *p* < 0.05 (2) UPDRS (gait scale): NS

BBS, Berg balance scale; Equistasi^®^, wearable proprioceptive stabilizer; GPR; Global PR, PR method based on the global postural reeducation concept; KT, Kinesio taping; NS, not significant; PR, postural rehabilitation; Su-Per, surfaces for perceptive rehabilitation; TUG, timed up and go; UPDRS, Unified Parkinson’s Disease Rating Scale.

## Data Availability

Data visualized in the graphs of this manuscript are available from the corresponding authors upon reasonable request.

## Abbreviations

The following abbreviations are used in this manuscript:

EQ-5D	EuroQol-5 Dimension;
MDS-UPDRS	Movement Disorder Society-Unified Parkinson’s Disease Rating Scale
NRS	numerical rating scale
ODI	Oswestry Disability Index
PD	Parkinson disease
QoL	quality of life
RCT	randomized controlled trial
ROM	range of motion
SVA	sagittal vertical axis
TK	thoracolumbar kyphosis

## Appendix A

**Table A1:** Contents of routine medication treatment.

Herbal Medicine	Compositions
Jaseng Gongjin-Dan	*Cervus nipponT., Aquilaria agallocha, Cornus officinalis, Angelica gigas Nakai*
Doinseunggi-tang	*Rheum palmatum, Cinnamomum cassia Blume, Glycyrrhiza uralensis, Erigeron canadensis L.*
Medication	Compositions
Perkin	100 mg 4.5T #3
Fosamax	70 mg 1T #1
Synthyroxine	1T #1
Tenelia M	10/500 mg 1T #1
Livalo	1T #1
Ganakhan	50 mg 1T #1 (Take PRN)
Phazyme	95 mg 1T #1 (Take PRN)
Stogar	10 mg 1T #1 (Take PRN)

**Table A2:** Bodyweight exercise checklist for patients.

Exercise Checklist			
Exercise			Illustrated Guide
Core muscle exercises	Supine position	Abdominal breathing exercise	
	Prone position	Deep erector spinae strengthening exercise	
		Gluteus maximus strengthening exercise	
Thoracic mobility exercises	Seated position	External oblique Abdominal muscle strengthening exercise	
		Serratus anterior strengthening exercise	

## References

[1] Ashour R., Jankovic J. (2006). Joint and skeletal deformities in Parkinson’s disease, multiple system atrophy, and progressive supranuclear palsy. Mov. Disord. Off. J. Mov. Disord. Soc..

[2] Baik J.S. (2016). Understanding of skeletal deformities in Parkinson’s disease. Indian J. Med. Res..

[3] Bissolotti L., Berjano P., Zuccher P., Zenorini A., Buraschi R., Villafañe J.H., Negrini S. (2017). Sagittal balance is correlated with Parkinson’s Disease clinical parameters: An overview of spinopelvic alignment on 175 consecutive cases. Eur. Spine J..

[4] Schrag A., Jahanshahi M., Quinn N. (2000). What contributes to quality of life in patients with Parkinson’s disease?. J. Neurol. Neurosurg. Psychiatry.

[5] Schrag A., Ben-Shlomo Y., Quinn N. (2002). How common are complications of Parkinson’s disease?. J. Neurol..

[6] Ganz D.A., Bao Y., Shekelle P.G., Rubenstein L.Z. (2007). Will my patient fall?. JAMA.

[7] van der Heeden J.F., Marinus J., Martinez-Martin P., Rodriguez-Blazquez C., Geraedts V.J., van Hilten J.J. (2016). Postural instability and gait are associated with severity and prognosis of Parkinson disease. Neurology.

[8] Rizzo G., Copetti M., Arcuti S., Martino D., Fontana A., Logroscino G. (2016). Accuracy of clinical diagnosis of Parkinson disease: A systematic review and meta-analysis. Neurology.

[9] Palakurthi B., Burugupally S.P. (2019). Postural instability in Parkinson’s disease: A review. Brain Sci..

[10] Lenoir T., Guedj N., Boulu P., Guigui P., Benoist M. (2010). Camptocormia: The bent spine syndrome, an update. Eur. Spine J..

[11] Sakai W., Nakane S., Urasaki E., Toyoda K., Sadakata E., Nagaishi A., Fukudome T., Yamakawa Y., Matsuo H. (2017). The cross-sectional area of paraspinal muscles predicts the efficacy of deep brain stimulation for camptocormia. J. Park. Dis..

[12] Babat L.B., McLain R.F., Bingaman W., Kalfas I., Young P., Rufo-Smith C. (2004). Spinal surgery in patients with Parkinson’s disease: Construct failure and progressive deformity. Spine.

[13] Chan A.K., Chan A.Y., Lau D., Durcanova B., Miller C.A., Larson P.S., Starr P.A., Mummaneni P.V. (2018). Surgical management of camptocormia in Parkinson’s disease: Systematic review and meta-analysis. J. Neurosurg..

[14] Kim Y.H., Lee J.M., Lee E.J., Oh M.S. (2017). Effects of Daoyin Exercise Therapy Combined with Korean Medicine Treatment on the Pain and Function Improvement of Low Back Pain Patients Diagnosed with Lumbar Disc Herniation: A Retrospective Observational Study. J. Physiol. Pathol. Korean Med..

[15] Terashi H., Endo K., Aizawa H. (2023). Characteristics of sagittal spinopelvic alignment in patients with Parkinson’s disease presenting with dropped head syndrome: A case series study. BMC Neurol..

[16] Uehara M., Takahashi J., Ikegami S., Tokida R., Nishimura H., Sakai N., Kato H. (2019). Sagittal spinal alignment deviation in the general elderly population: A Japanese cohort survey randomly sampled from a basic resident registry. Spine J..

[17] Goetz C.G., Poewe W., Dubois B., Schrag A., Stern M., Lang A. (2008). MDS-Unified Parkinson’s Disease Rating Scale (MDS-UPDRS). https://www.movementdisorders.org/MDS/MDS-Rating-Scales/MDS-Unified-Parkinsons-Disease-Rating-Scale-MDS-UPDRS.htm.

[18] Fairbank J.C., Pynsent P.B. (2000). The Oswestry disability index. Spine.

[19] Balestroni G., Bertolotti G. (2012). EuroQol-5D (EQ-5D): An instrument for measuring quality of life. Monaldi Arch. Chest Dis..

[20] Seo B.K. (2020). Korean Medicine Clinical Practice Guideline for lumbar disc herniation.

[21] Zhao Y., Zhang Z., Qin S., Fan W., Li W., Liu J., Wang S., Xu Z., Zhao M. (2021). Acupuncture for Parkinson’s disease: Efficacy evaluation and mechanisms in the dopaminergic neural circuit. Neural Plast..

[22] Kim S.-N., Doo A.-R., Park J.-Y., Bae H., Chae Y., Shim I., Lee H., Moon W., Lee H., Park H.-J. (2011). Acupuncture enhances the synaptic dopamine availability to improve motor function in a mouse model of Parkinson’s disease. PLoS ONE.

[23] Yan L., Zhang J., Wang X., Zhou Q., Wen J., Zhao H., Guo K., Zeng J. (2024). Efficacy of acupuncture for lumbar disc herniation: Changes in paravertebral muscle and fat infiltration–a multicenter retrospective cohort study. Front. Endocrinol..

[24] Kim D., Baek G.G., Shin B.-C. (2023). An Umbrella Review of Systematic Reviews for Chuna (or Tuina) Manual Therapy on Musculoskeletal Disorders. Perspect. Integr. Med..

[25] Capecci M., Serpicelli C., Fiorentini L., Censi G., Ferretti M., Orni C., Renzi R., Provinciali L., Ceravolo M.G. (2014). Postural rehabilitation and Kinesio taping for axial postural disorders in Parkinson’s disease. Arch. Phys. Med. Rehabil..

[26] Morrone M., Miccinilli S., Bravi M., Paolucci T., Melgari J.M., Salomone G., Picelli A., Spadini E., Ranavolo A., Saraceni V.M. (2016). Perceptive rehabilitation and trunk posture alignment in patients with Parkinson disease: A single blind randomized controlled trial. Eur. J. Phys. Rehabil. Med..

[27] Romanato M., Guiotto A., Spolaor F., Bakdounes L., Baldassarre G., Cucca A., Peppe A., Volpe D., Sawacha Z. (2021). Changes of biomechanics induced by Equistasi^®^ in Parkinson’s disease: Coupling between balance and lower limb joints kinematics. Med. Biol. Eng. Comput..

[28] Doherty K.M., Van De Warrenburg B.P., Peralta M.C., Silveira-Moriyama L., Azulay J.-P., Gershanik O.S., Bloem B.R. (2011). Postural deformities in Parkinson’s disease. Lancet Neurol..

[29] Schulz-Schaeffer W.J., Margraf N.G., Munser S., Wrede A., Buhmann C., Deuschl G., Oehlwein C. (2015). Effect of neurostimulation on camptocormia in Parkinson’s disease depends on symptom duration. Mov. Disord..

[30] Schulz-Schaeffer W.J. (2016). Camptocormia in Parkinson’s disease: A muscle disease due to dysregulated proprioceptive polysynaptic reflex arch. Front. Aging Neurosci..

[31] Chatilow L., DeLany J. (2002). Clinical Application of Neuromuscular Techniques: The Lower Body.

[32] Minakawa Y., Miyazaki S., Waki H., Yoshida N., Iimura K., Itoh K. (2022). Trigger point acupuncture and exercise for chronic low back pain in older adult: A preliminary randomized clinical trial. J. Acupunct. Meridian Stud..

[33] Castro-Sánchez A.M., Matarán-Penarrocha G.A., Arroyo-Morales M., Saavedra-Hernández M., Fernández-Sola C., Moreno-Lorenzo C. (2011). Effects of myofascial release techniques on pain, physical function, and postural stability in patients with fibromyalgia: A randomized controlled trial. Clin. Rehabil..

[34] Sun Y., Li L., Chen Y., Wang L., Zhai L., Sheng J., Liu T., Jin X. (2022). Feasibility and positive effects of scalp acupuncture for modulating motor and cerebral activity in Parkinson’s disease: A pilot study. NeuroRehabilitation.

[35] Yeo S., Lim S., Choe I.H., Choi Y.G., Chung K.C., Jahng G.H., Kim S.H. (2012). Acupuncture Stimulation on GB 34 Activates Neural Responses Associated with P arkinson’s Disease. CNS Neurosci. Ther..

[36] Chae Y., Lee H., Kim H., Kim C.H., Chang D.I., Kim K.M., Park H.J. (2009). Parsing brain activity associated with acupuncture treatment in Parkinson’s diseases. Mov. Disord..

[37] Gu H., Hong J., Wang Z., Chen J., Yuan F., Jiang Y., Yang Y., Luo M., Zhang Z., He B. (2024). Association of MRI findings with paraspinal muscles fat infiltration at lower lumbar levels in patients with chronic low back pain: A multicenter prospective study. BMC Musculoskelet. Disord..

[38] Lv Y., Dai D.-C., Jiang H.-N., Wang L. (2021). Effect of electroacupuncture on lumbar disc herniation with different multifidus fatty infiltration rates. Zhongguo Zhen Jiu = Chin. Acupunct. Moxibustion.

[39] Yang Q., Wang Y., Zhao C., Pang S., Lu J., Chan P. (2023). α-Synuclein aggregation causes muscle atrophy through neuromuscular junction degeneration. J. Cachexia Sarcopenia Muscle.

[40] Symons T.B., Park J., Kim J.H., Kwon E.H., Delacruz J., Lee J., Park Y., Chung E., Lee S. (2023). Attenuation of skeletal muscle atrophy via acupuncture, electro-acupuncture, and electrical stimulation. Integr. Med. Res..

[41] Song S.Y., No J.Y., Seol J.Y. (2023). A Case Report of Scalp Acupuncture and Chuna Manual Therapy for a Patient with Idiopathic Parkinson’s Disease with Walking Disorders due to Lower Extremity Rigidity. J. Intern. Korean Med..

[42] Saur P.M., Ensink F.-B.M., Frese K., Seeger D., Hildebrandt J. (1996). Lumbar range of motion: Reliability and validity of the inclinometer technique in the clinical measurement of trunk flexibility. Spine.

[43] Yao Y., Zhenni Z., Fengqin C., Yufei L., Xiangtian P., Xiao X., Zhiling S. (2023). Effectiveness of moxibustion alone on lumbar disc herniation: A Meta-analysis of randomized controlled trials. J. Tradit. Chin. Med..

[44] Chen J., Xu J., Huang P., Luo Y., Shi Y., Ma P. (2022). The potential applications of traditional Chinese medicine in Parkinson’s disease: A new opportunity. Biomed. Pharmacother..

[45] Mazhar T., Jameel A., Sharif F., Asghar M. (2023). Effects of conventional physical therapy with and without proprioceptive neuromuscular facilitation on balance, gait, and function in patients with Parkinson’s disease. J. Pak. Med. Assoc..

[46] Luan L., Zhu M., Adams R., Witchalls J., Pranata A., Han J. (2023). Effects of acupuncture or similar needling therapy on pain, proprioception, balance, and self-reported function in individuals with chronic ankle instability: A systematic review and meta-analysis. Complement. Ther. Med..

[47] Han J., Adams R., Waddington G. (2020). “Imposed” and “obtained” ankle proprioception across the life span—Commentary on Djajadikarta et al. J. Appl. Physiol..

